# Qualitative and Quantitative Detection of Botulinum Neurotoxins from Complex Matrices: Results of the First International Proficiency Test

**DOI:** 10.3390/toxins7124857

**Published:** 2015-11-25

**Authors:** Sylvia Worbs, Uwe Fiebig, Reinhard Zeleny, Heinz Schimmel, Andreas Rummel, Werner Luginbühl, Brigitte G. Dorner

**Affiliations:** 1 Biological Toxins, Centre for Biological Threats and Special Pathogens, Robert Koch Institute, Seestrasse 10, 13353 Berlin, Germany; worbss@rki.de (S.W.); fiebigu@rki.de (U.F.); 2 European Commission, Joint Research Centre, Institute for Reference Materials and Measurements, Retieseweg 111, 2440 Geel, Belgium; reinhard.zeleny@ec.europa.eu (R.Z.); heinz.schimmel@ec.europa.eu (H.S.); 3 toxogen GmbH, Feodor-Lynen-Strasse 35, 30625 Hannover, Germany; rummel@toxogen.de; 4 ChemStat, Aarstrasse 98, 3005 Bern, Switzerland; info@chemstat.ch

**Keywords:** proficiency test, BoNT, reference material, standardized detection

## Abstract

In the framework of the EU project EQuATox, a first international proficiency test (PT) on the detection and quantification of botulinum neurotoxins (BoNT) was conducted. Sample materials included BoNT serotypes A, B and E spiked into buffer, milk, meat extract and serum. Different methods were applied by the participants combining different principles of detection, identification and quantification. Based on qualitative assays, 95% of all results reported were correct. Successful strategies for BoNT detection were based on a combination of complementary immunological, MS-based and functional methods or on suitable functional *in vivo*/*in vitro* approaches (mouse bioassay, hemidiaphragm assay and Endopep-MS assay). Quantification of BoNT/A, BoNT/B and BoNT/E was performed by 48% of participating laboratories. It turned out that precise quantification of BoNT was difficult, resulting in a substantial scatter of quantitative data. This was especially true for results obtained by the mouse bioassay which is currently considered as “gold standard” for BoNT detection. The results clearly demonstrate the urgent need for certified BoNT reference materials and the development of methods replacing animal testing. In this context, the BoNT PT provided the valuable information that both the Endopep-MS assay and the hemidiaphragm assay delivered quantitative results superior to the mouse bioassay.

## 1. Introduction

Botulinum neurotoxins (BoNTs) comprise a family of high molecular weight bacterial toxins which are produced by the anaerobic Gram-positive bacteria *Clostridium (C.) botulinum*, *C. butyricum* and *C. baratii*. BoNTs are known as causative agents of the rare, but severe neurological disease botulism characterized by descending flaccid paralysis including double vision, ptosis, dyspnea, constipation and/or nausea and in severe cases death by respiratory failure [1]. The disease occurs in three major forms: Food-borne botulism is caused by ingestion of food contaminated with BoNT. Wound botulism occurs after uptake of *C. botulinum* spores into wounds and subsequent germination with parallel production of BoNT. Finally, infant botulism is caused in babies within their first year of life by colonization of the intestinal tract and toxin production [1]. While *C. botulinum* is principally able to produce up to four different types of toxins, botulinolysin (a pore-forming toxin), C2 and C3 toxin (ADP-ribosylating toxins) and the neurotoxin, only the latter one is linked to botulism [2]. BoNTs are remarkable in different ways:

(i) *The high variability of BoNTs*. The group of BoNTs can be distinguished into seven confirmed serotypes, A through G. While serotypes A, B, E, and F cause botulism in humans, serotypes C and D have been attributed to veterinary botulism [3]. The last decade has shown that there is a previously unrecognized variability among BoNT molecules: the serotypes A, B, E, and F can be distinguished into more than 40 subtypes based on their amino acid sequence, antibody binding and functional activity. Depending on the serotype, the sequence variability among subtypes can reach 36% on amino acid level [4,5,6,7,8,9,10,11]. Additionally, mosaic toxins have been described [12,13,14].
(ii) *Complex formation of BoNTs*. In acidic bacterial supernatants the neurotoxins are not found as pure 150 kDa di-chain toxins, but are associated with non-toxic non-hemagglutinin (NTNHA), and additionally—depending on serotype and subtype—with up to three different hemagglutinins (HA17, HA33 and HA70) constituting different high molecular weight progenitor toxin complexes (PTCs): M-PTC is composed of BoNT plus a corresponding NTNHA forming an interlocked complex of 290 kDa [15] and is produced by all strains. The L-PTC consisting of BoNT, NTNHA, HA70, HA17, and HA33 at a stoichiometry of 1:1:3:3:6 resulting in a 760 kDa molecule [16,17] is found in strains producing serotypes A, B, C, D and G. While the exact mechanism of BoNT uptake is currently under investigation, it has already been shown that the complex proteins play a role in stabilizing the toxin during the passage through the gastrointestinal tract (NTNHA) and in the adsorption process at intestinal epithelia (HA proteins) [15,18,19,20,21]. On the genetic level, the genes encoding for BoNT and the different complex proteins are arranged in a bicistronic gene cluster comprising an *ntnha-bont* operon and an *ha* operon (*ha^+^orfX^–^* cluster; [4]). Apart from this cluster, a second gene cluster is observed, where the genes encoding HA are replaced by three genes encoding OrfX proteins of yet unknown expression and function (*ha^–^orfX^+^* cluster [4,19]).
(iii) *The exquisite toxicity of BoNTs*. The BoNTs constitute the most poisonous toxins known today. Lethal amounts of crystalline BoNT/A L-PTC in humans are estimated from primate studies to be 1 μg/kg body weight when taken orally, 10 ng/kg by inhalation and 1 ng/kg intravenously or intramuscularly [22].
(iv) *The highly specific mechanism of intoxication*. After oral uptake, BoNTs reach the neuromuscular junction of cholinergic neurons. As members of the family of A–B protein toxins the BoNT molecules consist of a 100 kDa B-domain called heavy chain (HC) and an enzymatically active 50 kDa A-domain called light chain (LC) which are covalently linked by a disulfide bridge [23]. The HC confers receptor binding and cellular uptake, while the LC acts as zinc-dependent metalloprotease that specifically cleaves proteins of the soluble *N*-ethylmaleimide-sensitive factor attachment protein receptor (SNARE) complex which mediates the release of acetylcholine from synaptic vesicles. The SNARE complex is formed by the assembly of the proteins synaptosome-associated protein of 25 kDa (SNAP-25), syntaxin and vesicle-associated membrane protein (VAMP)/synaptobrevin [24]. While BoNT/A, C and E cleave at different sites of SNAP-25, BoNT/C also targets syntaxin. BoNT/B, D, F and G cleave at distinct sites of VAMP. After cleavage of any of the above-mentioned SNARE proteins, the formation of the SNARE complex is inhibited, resulting in the blockage of neurotransmitter release. This leads to the classical paralytic symptoms of botulism [25,26].

BoNTs are typical dual-use substances: as the most toxic substances known to man and as the causative agents of botulism the toxins attracted attention of those intending criminal, terroristic and military misuse. Exemplarily, the Japanese Aum cult tried to disperse *C. botulinum* culture supernatants in Tokyo on different occasions between 1990 and 1995 [27]. Additionally, BoNT was included in different weapons programs during World War II and later [28]. Based on its history, BoNT is a prohibited substance under the Biological Weapons Convention (BWC) and classified as select agent category A by the Centers for Disease Control and Prevention (CDC, Atlanta, GA, USA). On the positive side, based on the highly specific biological action *in vivo*, BoNTs are being applied medically as long-acting peripheral muscle relaxant with annual revenues of more than $2.5 billion. Mainly serotype A and to a limited extent serotype B have developed into an indispensable pharmaceutical agent and are currently approved for the treatment of more than 20 neurological and non-neurological diseases, among them strabismus, dystonia, spasticity, hyperhidrosis and migraine [29]. Additionally, among the general public BoNT has become famous for its application in aesthetic medicine [30].

Considering the different aspects of security, health and consumer protection as well as medical application the rapid detection, precise identification and accurate potency determination of BoNT is important. Generally, the different fields of BoNT research have quite contrary requirements for diagnostic approaches: while in the case of botulism diagnostics from clinical, food and environmental samples the focus is on the reliable detection of all serotypes and subtypes—including known and unknown subtypes—in complex matrices, highly purified pharmacological products require the precise and statistically valid potency determination [31]. Different functional, immunological, spectrometric and molecular methods have been developed to cope with the different technical demands [31,32]. Still, in many application fields the classical mouse bioassay (MBA) introduced in the 1920s [33] is considered as a “gold standard method” since it has an exquisite detection limit (down to a few pg toxin per mouse), measures all steps of BoNT’s mode of action and is able to detect all functionally active BoNT variants; therefore it is currently included in official methods and national guidelines (e.g., AOAC Official Method 977.26 or the German Standard DIN 10102). Due to serious ethical concerns and a number of experimental disadvantages, replacement methods have been introduced, among them the mouse phrenic nerve hemidiaphragm (MPN) assay as an *ex vivo* method [34,35]. In this type of assay an explanted preparation of the phrenic nerve connected with the hemidiaphragm muscle is electrically stimulated and the resulting muscle twitches are measured. Upon addition of BoNT, the time required to decrease the amplitude to 50% of the starting value is measured as paralytic half-time which is directly proportional to the dose of BoNT applied [36,37]. Additionally, *in vitro* activity assays measuring the catalytic endopeptidase activity of the LC of BoNT have been developed. Basically, endopeptidase assays display the serotype-specific proteolytic cleavage of SNARE proteins in conjunction with technically different read-outs, e.g., mass spectrometric (Endopep-MS assay [38]) or immunological detection of cleavage products (Endopep-ELISA [39,40]). In order to apply the enzymatic assay on real sample materials, an immunoaffinity enrichment step is usually applied, where the toxin is captured from the matrix using antibody-coated magnetic microbeads prior to performing the endopeptidase reaction, resulting in assay sensitivities similar or better to those of the MBA [31,41]. Endopeptidase assays usually detect the LC activity only. Therefore in different approaches HC-specific antibodies or alternatively receptor binding have been applied for toxin extraction and combined with detection of cleaved substrates [42,43,44]. Independent of the aforementioned approaches different cell-based assays have been developed which are currently in use for precise potency determination of highly pure pharmacological preparations and for BoNT inhibitor screening [45,46]. Their applicability for routine diagnostic purposes has to be evaluated in the future.

In contrast to *in vivo*, *ex vivo* or *in vitro* activity assays, pure immunological approaches detect only the presence of the toxin, not its activity. Nonetheless, sandwich enzyme-linked immunosorbent assays (ELISAs) are by far the most commonly employed *in vitro* method for BoNT detection due to their generally high sensitivity, their simplicity, short assay times and robust assay performance [31,47,48,49,50]. However, quality and validity of obtained data strictly depend on the quality and combination of the antibodies used. The same is true for immunochromatographic lateral flow assays (LFA) which are hand-held devices based on a sandwich ELISA performed on paper strips. Usually, they have restricted sensitivity compared to conventional ELISAs and are more prone to matrix interferences [31]. A number of interesting new developments have been published in recent years which aim at miniaturizing BoNT detection on the protein or functional level, among them microarray or biosensor applications, centrifugal microfluidic disk platforms and portable devices [51,52,53,54,55].

In light of the range of technical approaches, different analytical tools, sample preparation strategies and assay protocols used in expert laboratories, any comparison of experimental results is generally difficult. Additionally, no certified reference material is available, so that expert laboratories currently use either in-house purified BoNT standards or different commercial products of variable quality. Though different technologies are available in expert laboratories, no proficiency testing (PT) schemes or ring trials on dedicated techniques are available to identify good analytical strategies for the analysis of real samples. To the best of our knowledge, only one international exercise was previously organized that focused on highly pure pharmaceutical-grade BoNT preparations and specifically addressed potency testing by MBA [56]. With respect to the toxin-producing organism, an international exercise addressed the detection and typing of BoNT-producing Clostridia by real-time polymerase chain reaction [57].

Against this background, the current work has been undertaken in the framework of the EU-project EQuATox (Establishment of quality assurance for the detection of biological toxins of potential bioterrorism risk [58]) funded under the European Community’s Seventh Framework Programme to provide an overview and evaluation of existing methods for detection, identification and quantification of BoNTs on the protein level from real sample materials. In planning an appropriate exercise, it had to be considered that the current PT was the very first international activity testing the laboratories’ diagnostic capabilities, so it had to cover a broad range of potential technical approaches and capabilities. Along this line, the very first international exercise on BoNT detection and quantification was a “confidence building exercise” offering the possibility to discuss on potential difficulties and limitations with experts from different countries.

To this end, a PT scheme was designed in line with internationally accepted standards which comprised 13 test samples containing highly pure, recombinantly expressed BoNT/A, B or E [59] spiked into buffer, milk, meat extract and serum in a concentration range of 0.5–1000 ng/mL. Stability and homogeneity was tested according to Thompson *et al.* and ISO/IEC 17043:2012 [60,61]. For participating laboratories, the task was to qualitatively detect BoNT-containing samples—either with or without serotyping—by any technical approach available in the individual laboratory and, if possible, to quantify the toxin using own in-house reference materials. Here we provide an overview of qualitative and quantitative results obtained by 23 international expert laboratories from the health, food and security sector, among them 18 laboratories from EU countries. The results set a basis for further steps in quality assurance and highlight next priorities in the process of harmonization and standardization of analytical approaches.

## 2. Results and Discussion

### 2.1. Preparation of the BoNT Proficiency Test

To set up a proper PT test plan, 13 samples were selected for further preparatory analysis taking into account the following:

(i) The samples were planned to comprise the most important BoNT serotypes pathogenic to humans, *i.e.*, BoNT/A, BoNT/B and BoNT/E, to test the laboratories’ ability to detect and to differentiate the different neurotoxins (serotyping). BoNT/F was not considered further as PT material in this very first PT due to the rareness of natural BoNT/F cases and concerns regarding the stability of recombinant BoNT/F material. From all the different subtypes of serotypes described so far, subtypes BoNT/A1, BoNT/B1 and BoNT/E1 were chosen as representative subtypes and used in this study (abbreviated BoNT/A, B and E throughout this manuscript). Additionally, the 150 kDa neurotoxins as smallest, but fully functional and well defined entity representing the causative agent of botulism were used in this first PT. The purity of activated recombinant BoNT/A, BoNT/B and BoNT/E was determined to be ≥99% for BoNT/A, ≥96% for BoNT/B and ≥93% for BoNT/E, respectively (for details see [59]). To test for serotyping capabilities, BoNT/A, BoNT/B and BoNT/E were included as samples in the PT at an intermediate concentration (10 ng/mL) in 0.1% BSA/PBS (Table 1).
(ii) The samples needed to be detectable with a range of different techniques, as the PT was open with respect to the methods applied by the participants. The expectation of a technically open PT was to obtain information on best analytical practices. To this end, four different concentrations of purified BoNT/A spiked into buffer containing a stabilizing protein (BSA) were selected: a very high (1000 ng/mL), a high (100 ng/mL), an intermediate (10 ng/mL) and a moderate (0.5 ng/mL) concentration of recombinantly expressed, highly purified BoNT/A in 0.1% BSA/PBS (Table 1).
(iii) In order to analyze the influence of complex matrices on the detection of BoNT, the intermediate concentration of BoNT/A (10 ng/mL) was spiked into human serum, semi-skimmed milk and a particle-free, sterile extract of minced meat. Both food matrices (semi-skimmed milk and meat extract) were also spiked with the high concentration (100 ng/mL) of BoNT/A, assuming that in food poisoning the toxin concentration would be higher.
(iv) Finally, a complex sample was set up comprising both BoNT/A and BoNT/B at equal concentrations (5 ng/mL each) in 0.1% BSA/PBS. This sample mimicked naturally occurring *C. botulinum* strains that produce two or three different BoNTs [62].

**Table 1:** Proficiency test (PT) test plan.

Samples Selected as Potential PT Samples for Further Stability Testing		PT Sample Number
1	1000 ng/mL of BoNT/A in 0.1% BSA/PBS	S12
2	100 ng/mL of BoNT/A in 0.1% BSA/PBS	S7
3	10 ng/mL of BoNT/A in 0.1% BSA/PBS	S2
4	0.5 ng/mL of BoNT/A in 0.1% BSA/PBS	S9
5	100 ng/mL of BoNT/A in semi-skimmed milk	S13
6	10 ng/mL of BoNT/A in semi-skimmed milk	S10
7	100 ng/mL of BoNT/A in meat extract	S5
8	10 ng/mL of BoNT/A in meat extract	S1
9	10 ng/mL of BoNT/A in human serum	S11
10	5 ng/mL of BoNT/A and 5 ng/mL of BoNT/B in 0.1% BSA/PBS	S8
11	10 ng/mL of BoNT/B in 0.1% BSA/PBS	S6
12	10 ng/mL of BoNT/E in 0.1% BSA/PBS	S4
13	0.1% BSA/PBS	S3

**Figure 1:**
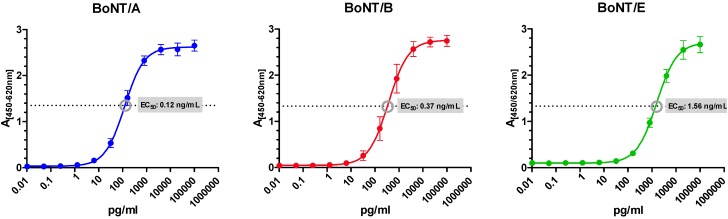
Sandwich enzyme-linked immunosorbent assays (ELISAs) specifically detecting BoNT/A, BoNT/B or BoNT/E. The ELISAs were performed as described in the Experimental Section and were used to perform the stability and homogeneity studies as well as for determination of the assigned concentrations of the PT samples. The curves show results of three to five independent measurements performed in duplicate using the recombinant toxins produced in [59]. EC_50_ values indicating the half-maximum concentration that can be measured by the given ELISAs are indicated by a gray circle.

The potential PT samples as depicted in Table 1 were further analyzed by stability testing. According to Thompson *et al.* and ISO/IEC 17043:2012, samples have to be sufficiently stable during the predefined testing period which was set to four weeks by the PT organizer [60,61]. Stability testing was performed by sandwich ELISAs specifically detecting BoNT/A, BoNT/B or BoNT/E, respectively. Corresponding standard curves of the ELISAs used for the three analytes are depicted in Figure 1.

**Figure 2:**
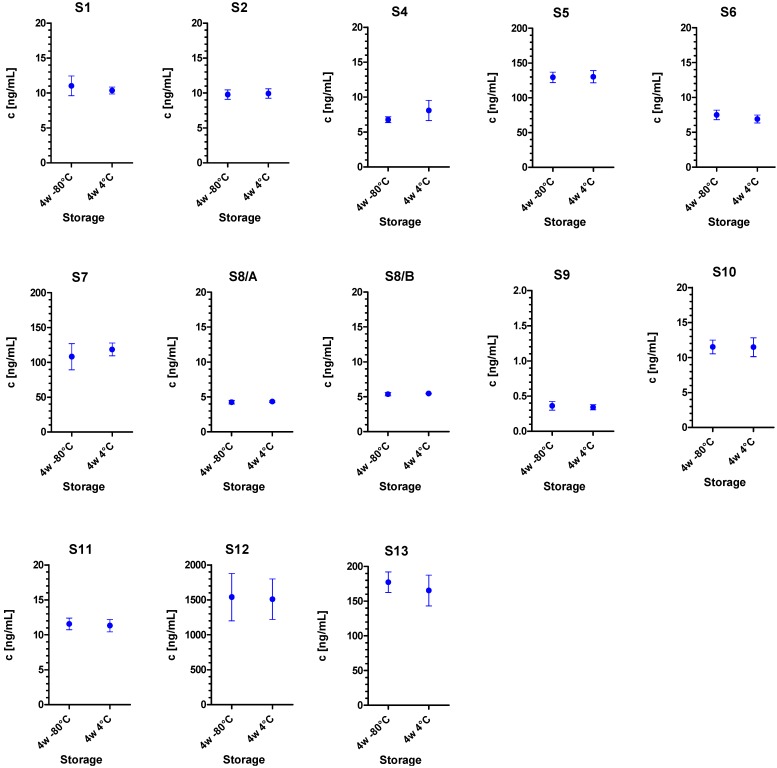
Stability of PT samples as measured by BoNT-specific sandwich ELISAs. All 13 samples were either stored at −80 °C or 4 °C for four weeks (4w −80 °C or 4w 4 °C). Concentrations in samples S1, S2, S4, S5, S7, S8, S9, S10, S11, S12 and S13 were quantitated by BoNT/A-specific ELISA, samples S6 and S8 by BoNT/B-specific ELISA and S4 by BoNT/E-specific ELISA; error bars indicate the standard deviations obtained for five randomly selected sample replicates per storage condition.

For precise quantification, all BoNT-containing samples were diluted to a concentration close to the EC_50_ value which is the point of highest precision in measurement (EC_50_[BoNT/A] = 0.12 ng/mL; EC_50_[BoNT/B] = 0.37 ng/mL; EC_50_[BoNT/E] = 1.56 ng/mL; Figure 1). In order to demonstrate sample stability during the PT test period, 10 aliquots of each sample S1 to S13 were prepared and analyzed for stability: five aliquots were stored for four weeks at −80 °C, and five aliquots were stored for four weeks at 4 °C for comparison. All sample sets were analyzed simultaneously on a single day by the different BoNT ELISAs corresponding to the serotype contained in the respective sample. As indicated in Figure 2, experimental results generally showed a suitable stability of all samples S1 to S13 at 4 °C over a period of four weeks. This result was confirmed statistically by Dunnett’s test which showed no significant deviation in concentrations under these two storage conditions (*p* > 0.05; not shown).

**Figure 3:**
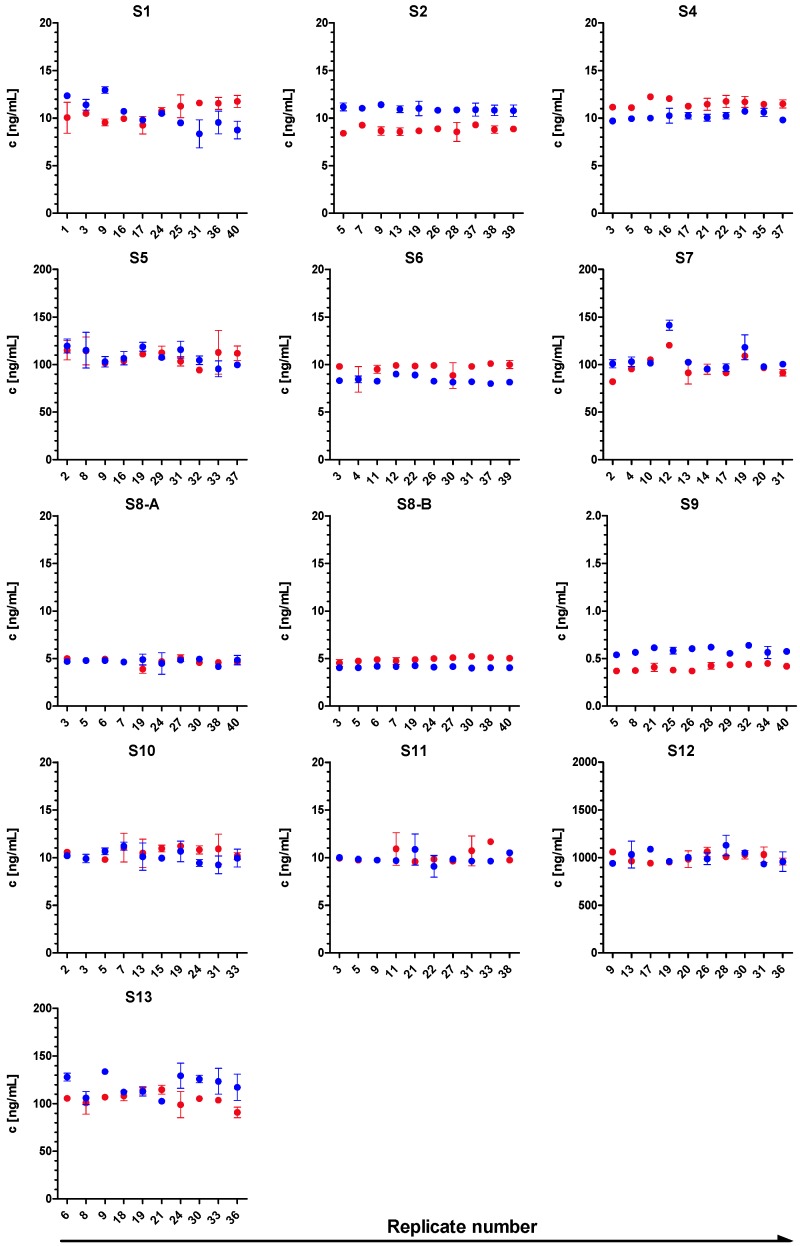
Homogeneity study. Ten randomly selected test portions of each sample (S1–S13) were analyzed by BoNT/A-, BoNT/B- or BoNT/E-specific ELISA, respectively, in two independent experiments (depicted in red and blue), each performed in duplicate. The mean concentration of each duplicate ± standard deviation (error bars) was plotted against the 10 replicates of each sample.

Based on the stability study, samples S1 to S13 were selected as suitable PT samples. For the actual PT, 40 aliquots of each sample S1 to S13 were prepared as before. Of these, 10 aliquots of each sample were randomly selected for homogeneity testing. Homogeneity of each test material was assessed according to Thompson *et al.* [60] and ISO/IEC 17043:2012 [61] by employing the corresponding sandwich ELISAs and deducing the concentration of the 10 test portions of each sample. Figure 3 graphically displays the results of the homogeneity tests in which 10 aliquots of each sample were quantified twice in duplicate by ELISA. At first glance sufficient homogeneity was observed. In some cases the variance between the duplicates was larger than the variance between the two experiments or the 10 replicates. Statistically, Cochran tests showed no outlying variance for all samples at a significance level of 0.05 (not shown).

Though the highly purified recombinant BoNT/A, BoNT/B and BoNT/E preparations, thoroughly characterized by Weisemann *et al.* [59] and used here to spike PT samples, represent well-defined, qualified materials, they are still no certified reference materials. According to Thompson *et al.*, in this situation it is necessary to determine the protein concentration experimentally after spiking the purified toxins into the buffer or matrix [60]. This provides the “nominal concentration” of the samples as opposed to the “theoretical concentration” that is the known spiked concentration, assuming there are no losses during sample preparation, no matrix effects or other disturbing factors.

To this end, the experimental data obtained in the homogeneity testing for the 10 randomly selected aliquots were used to determine the nominal concentration of the PT samples. For each randomly selected aliquot one dilution close to the EC_50_ value of the corresponding ELISA for BoNT/A, BoNT/B or BoNT/E, respectively, was chosen to calculate the nominal concentrations. The dilutions were measured in duplicate in two independent experiments. The calculated concentrations of the ten replicates of each sample measured in two experiments were statistically analyzed, and estimates of the nominal concentrations were obtained with the robust algorithm according to ISO 5725-5:1998 [63]. The robust means of the nominal concentrations were adopted as “assigned values” for the evaluation of the quantitative PT results reported by the participants. Table 2 summarizes the theoretical concentration for each sample, the robust estimate of the mean nominal concentration based on the experiments performed in the organizer’s laboratory as well as the robust estimate of the standard deviation of the nominal concentrations. Additionally, for the subsequent quantitative analysis of PT results reported by the participants, the standard deviation for proficiency assessment σ_p_ was calculated as given in the Experimental Section.

**Table 2:** Proficiency test: sample identity and statistics.

Sample	Matrix	Serotype/Measurand	*c*(theoretical) * (ng/mL)	*c*(nominal) ** (ng/mL)	σ(rob) (ng/mL)	σ_p_ (ng/mL)
S1	Meat extract	BoNT/A	10	10.5	1.4	2.68
S2	0.1% BSA/PBS	BoNT/A	10	9.9	1.3	2.52
S3	0.1% BSA/PBS	–	–	–	–	–
S4	0.1% BSA/PBS	BoNT/E	10	10.9	0.9	2.77
S5	Meat extract	BoNT/A	100	108.0	10.0	27.60
S6	0.1% BSA/PBS	BoNT/B	10	9.0	0.9	2.29
S7	0.1% BSA/PBS	BoNT/A	100	100.0	9.0	25.50
S8/A	0.1% BSA/PBS	BoNT/A	5	4.7	0.3	1.21
S8/B	0.1% BSA/PBS	BoNT/B	5	4.5	0.5	1.15
S9	0.1% BSA/PBS	BoNT/A	0.5	0.5	0.1	0.13
S10	Milk	BoNT/A	10	10.3	0.8	2.64
S11	Serum	BoNT/A	10	9.8	0.3	2.51
S12	0.1% BSA/PBS	BoNT/A	1000	1001.0	69.0	255.00
S13	Milk	BoNT/A	100	112.0	13.0	28.50

* The “theoretical concentration” was the known concentration of BoNT/A, BoNT/B or BoNT/E that was spiked into the different matrices; ** Robust estimates of mean nominal concentrations as determined experimentally by the organizing laboratory by ELISA for BoNT/A, BoNT/B or BoNT/E, respectively. Adopted as “assigned values” for the evaluation of the quantitative PT results; σ(rob): robust estimate of the standard deviation of the nominal concentrations; σ_p_: standard deviation for proficiency assessment.

Determination of the nominal concentrations of the 13 samples concluded the preparatory experimental part of the BoNT PT. With respect to shipment of active toxin-containing samples and depending on the destination of the shipment, different individual authorizations were required and obtained by the national authorities of the participating countries, e.g., clearance certificates and import or export permits. The actual shipment was realized using a dedicated shipper (World Courier, Berlin, Germany) as security transport: the transport of toxins as a dangerous goods shipment followed the classification toxic class 6.1, packaging group PGI (P652), UN3172. The material was packed in IATA/ADR-approved 4GU boxes (Bio-Bottles, Alex Breuer GmbH, Cologne, Germany), and the dispatch of samples from the organizer’s laboratory was done by a certified shipping agent. The samples were transported in Bio-Bottles securely locked in 20 kg steel containers equipped with temperature loggers and cooling devices and were tracked throughout the shipment.

Two months before the actual shipment of samples, the interested laboratories obtained an official announcement letter including a nomination form and information on objectives of the PT, the test design, the potential sample materials and measurands, a timeline for the PT, basic information on reporting and analysis as well as comments on the requirements and regulations to be obeyed. Deadline to deliver results was announced to be four weeks after shipment of samples. All in all, 23 expert laboratories from 14 countries worldwide actively took part in the exercise and received 1 mL of samples S1 to S13. All samples reached their destination within three days at the latest. The electronically transmitted temperature logging files indicated that all packages arrived at their destinations at temperatures below 7 °C. The participants confirmed that all samples arrived cooled and in a good condition.

In order to re-confirm sample stability, the organizer’s laboratory measured once again the concentration in all samples in one randomly selected sample set four weeks after sample shipment (not shown). The concentrations determined in the post stability test were compared to the nominal concentrations determined before sending samples to PT participants in the homogeneity study and confirmed the findings of the pre PT stability studies, *i.e.*, that all samples were sufficiently stable during the period of the BoNT PT.

### 2.2. Results of the BoNT Proficiency Test

Since one major goal of the BoNT PT was to define good analytical strategies, the PT was open with respect to the methods applied by the PT participants. The participants were asked to deliver their results both qualitatively and/or quantitatively in two technically independent replicates (including all steps of sample preparation) per method applied, using a dedicated Excel reporting file. Additionally, since laboratories were completely free to combine different methods, analytical tools and approaches, they were asked to fill in a mandatory summary report summarizing their sample-specific conclusions in a final result sheet, taking into account different results that might have been obtained by applying different methods. One challenge in this first international PT was the restricted sample volume provided (1 mL), considering that in real botulism cases often the sample material—especially from clinical specimens—is restricted. So if a laboratory was planning to apply both qualitative and quantitative analysis or to combine different technical approaches, the volume per analysis had to be carefully planned. Qualitative and quantitative results reported by the PT participants were summarized in anonymized form and will be discussed in the next two sections. Any information on how many assays and exactly which assays were used by each laboratory was considered of potential dual-use-interest and therefore is not given here.

#### 2.2.1. Qualitative Results of the BoNT Proficiency Test

A variety of methods were applied in the BoNT PT, combining different principles of detection, identification and quantification. Qualitative results were assessed according to the degree of trueness of the participant’s assignments and color and number codes were used to indicate the assessment. For analysis and visualization of results, the qualitative results reported by the participants were color-coded and—when comparing individual methods—additionally number-coded according to the following legend:

(1)   correct positive (1), correct negative (−1), with serotyping(2)   correct positive (1), correct negative (−1), without serotyping(3)   false positive (10), false negative (−10)(4)   one of two reported results false positive (4.5) or false negative (−4.5)(5)   No response or not analyzed

**Figure 4:**
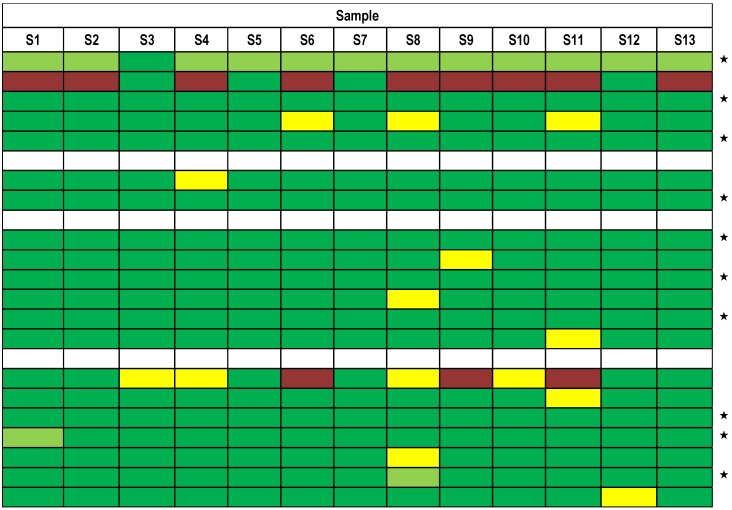
Summarized results reported by all 23 participating laboratories for samples S1–S13. The individual laboratories—each indicated in a horizontal row—were asked to summarize their experimental results for the 13 samples obtained by different methods. Qualitative results reported by the participants were color-coded according to the following legend: “correct, with serotyping of BoNT” (green) and “correct, without serotyping of BoNT” (light green); “false positive or false negative” (red); “one of two reported results false positive or false negative” (yellow); “no response or not analyzed” (white). Participating laboratories marked by an asterisk (★) delivered qualitatively correct results for all 13 samples, not taking into account serotyping of samples. Three participants did not summarize their results in the mandatory summary report; therefore they are given as a white row.

Qualitative results were submitted by 21 out of 23 participants; five participants used different technical approaches and reported results obtained by different techniques individually while the majority of all participating laboratories used preferentially a single technology to work on the 13 samples. Figure 4 displays an overview of all results reported in the mandatory summary and shows that, based on the assessment of qualitative results reported, 95% of all results reported were correct, corresponding to the majority of results given in dark green.

The high percentage of qualitatively correct results can be taken as an indication of a successfully conducted exercise, considering the fact that the PT was the first international one on BoNT detection. Each horizontal row in Figure 4 represents the summarized results of one participating laboratory. Twenty participants reported qualitative results including serotyping, and one participant reported presence of BoNT without serotyping (light green, Figure 4). Three participants did not summarize their results in the mandatory summary report; therefore they are given as a white row in Figure 4. The figure shows that 10 out of 23 laboratories provided correct results for all 13 samples, not taking into account their serotyping capabilities.

In order to extract relevant technical information from the qualitative measurements performed in the BoNT PT, the results reported after using different methods were further analyzed according to different parameters:

(i) based on sample identity comparing results provided for corresponding samples (e.g., comparison of results submitted for a given concentration of BoNT in buffer, milk, meat extract and serum; comparison of results given for different concentrations or different serotypes);
(ii) based on the comparison of different technological approaches (*i.e.*, comparison of different approaches like mouse bioassay, MS-based approaches or different ELISA assays displayed against each other).

**Figure 5:**
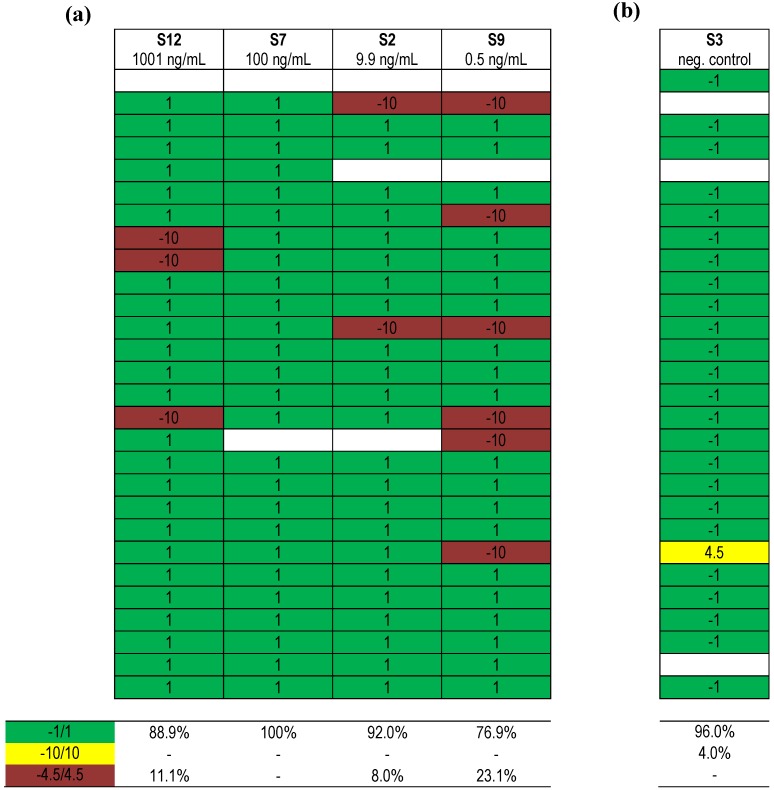
Qualitative results obtained for samples containing BoNT/A in different concentrations in buffered solution. (**a**) BoNT/A concentrations present in samples S12, S7, S2 and S9 are indicated in the table header; (**b**) Results obtained for negative control sample. Each row indicates a qualitative result reported after using a particular method; several laboratories reported more than one method. Qualitative results reported by the participants were color-coded as indicated above, with green corresponding to “correct positive (1) or correct negative (−1)”; yellow corresponding to “one of two reported results false positive (4.5) or false negative (−4.5)”; red corresponding to “false positive (10) or false negative (−10)” and white “no response or not analyzed”. Indicated below is the percentage of qualitative results reported for the given samples as “BoNT/A” (**a**) or “BoNT absent” (**b**).

**Figure 6:**
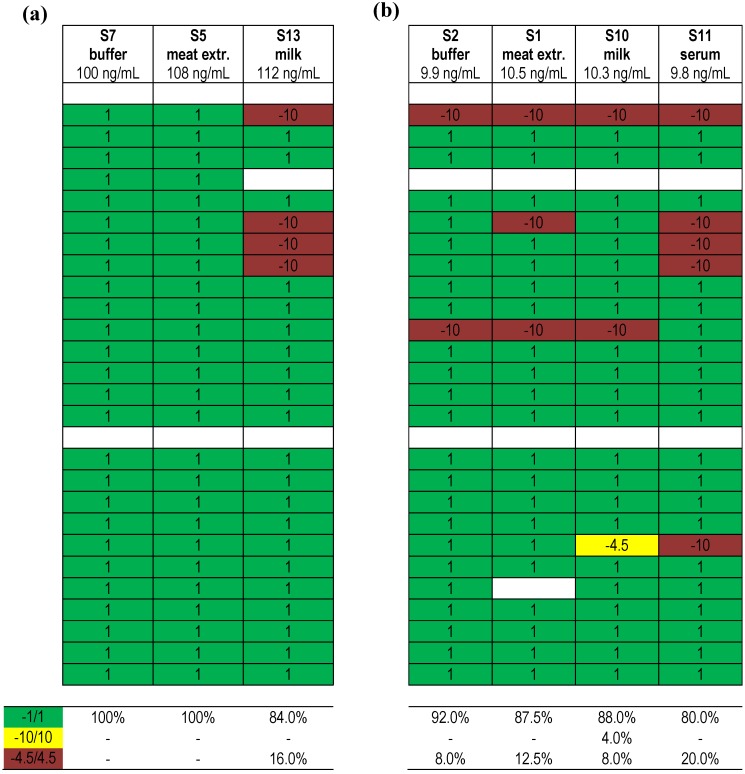
Qualitative results obtained for samples containing BoNT/A in different complex matrices. (**a**) Results obtained for samples containing ~100 ng/mL of BoNT/A spiked into complex matrices or into buffer; (**b**) Results obtained for samples containing ~10 ng/mL of BoNT/A spiked into complex matrices or into buffer. Each row indicates a qualitative result reported after using a particular method; several laboratories reported more than one method. Qualitative results reported by the participants were color-coded as described in Figure 5. Indicated below is the percentage of qualitative results reported for the given samples as “BoNT/A”.

**Figure 7:**
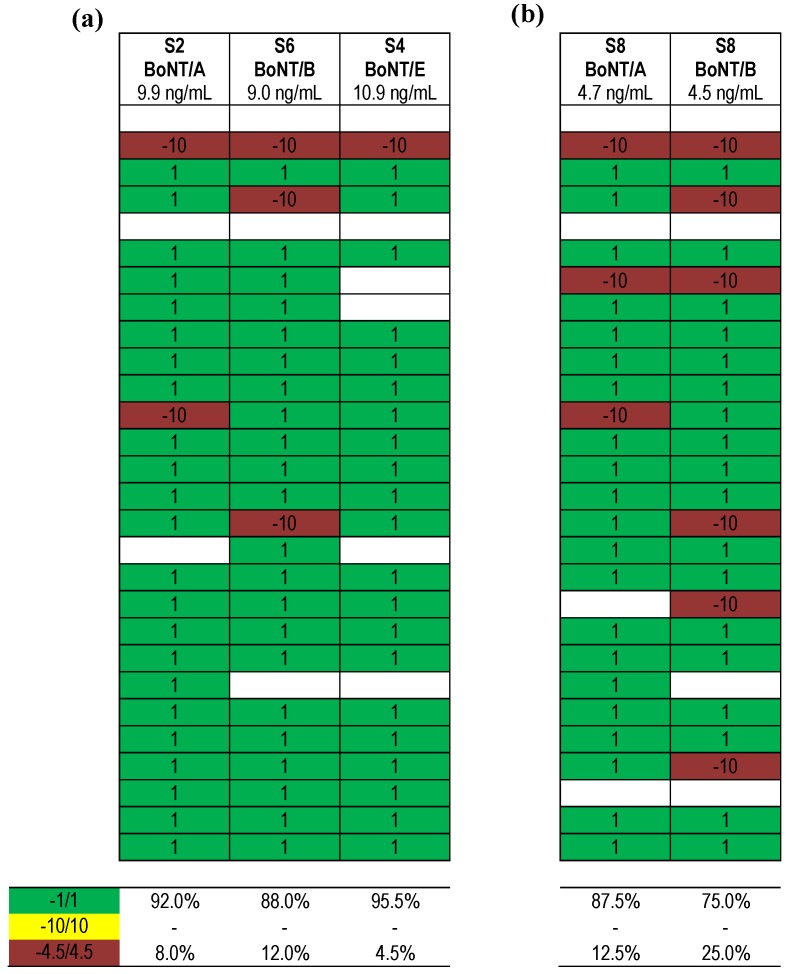
Qualitative results obtained for samples containing different BoNT serotypes. (**a**) Results obtained for samples containing different BoNT serotypes at ~10 ng/mL; (**b**) Results obtained for sample S8 containing two serotypes (BoNT/A and BoNT/B) in equal concentrations (~5 ng/mL each). Each row indicates a qualitative result reported after using a particular method; several laboratories reported more than one method. Qualitative results reported by the participants were color-coded as described in Figure 5. Indicated below is the percentage of qualitative results reported for S2 as “BoNT/A”, S6 as “BoNT/B”, S4 as “BoNT/E” and S8 as “BoNT/A” and “BoNT/B”.

**Figure 8:**
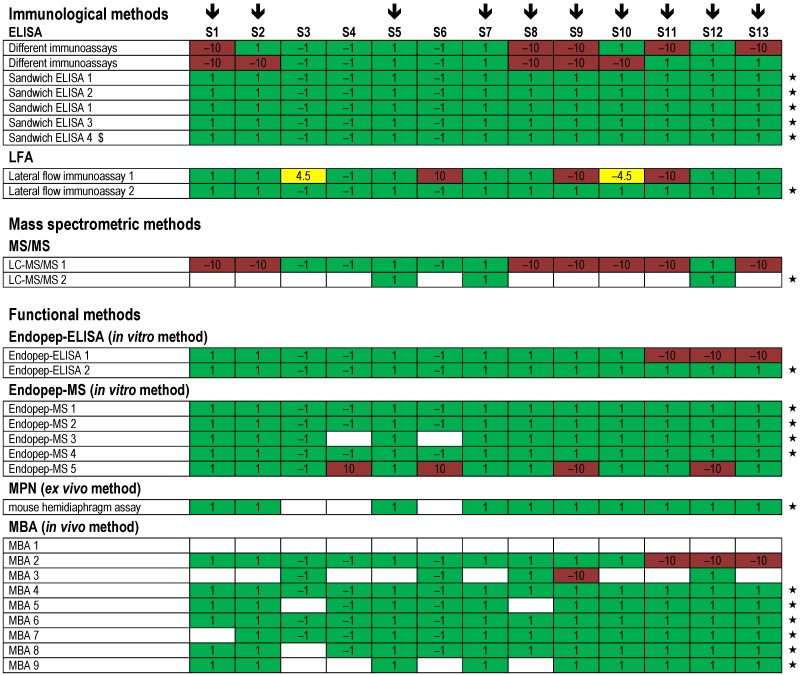
Qualitative results reported as “BoNT/A” for all 13 samples displayed by the different methods used. Samples containing BoNT/A are marked with a black arrow (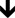). Each row indicates a qualitative result reported after using a particular method; several laboratories reported more than one method. Qualitative results reported by the participants were color-coded as described in Figure 5. Methods marked by an asterisk (★) delivered correct results for the samples analyzed. $, results have been taken from the laboratory’s quantitative reporting since they have, accidentally, not been reported qualitatively.

**Figure 9:**
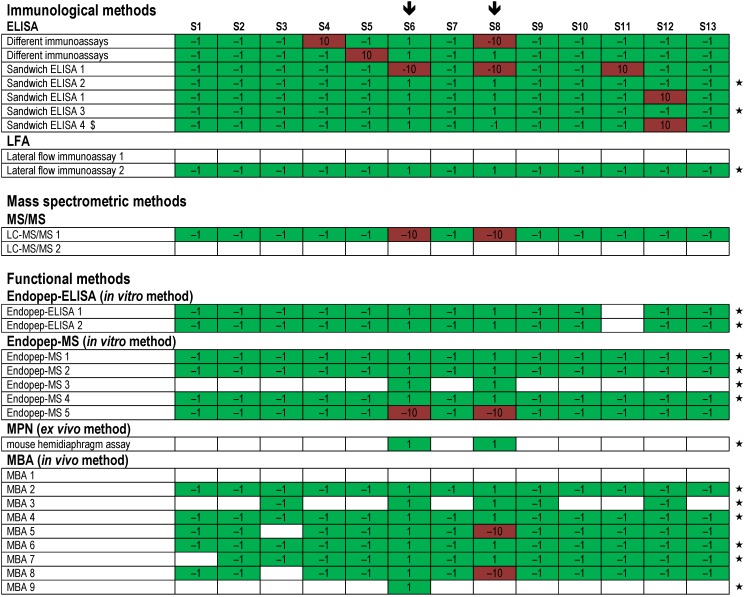
Qualitative results reported as “BoNT/B” for all 13 samples displayed by the different methods used. Samples containing BoNT/B are marked with a black arrow (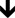). Each row indicates a qualitative result reported after using a particular method; several laboratories reported more than one method. Qualitative results reported by the participants were color-coded as described in Figure 5. Methods marked by an asterisk (★) delivered correct results for the samples analyzed. $, results have been taken from the laboratory’s quantitative reporting since they have, accidentally, not been reported qualitatively.

**Figure 10:**
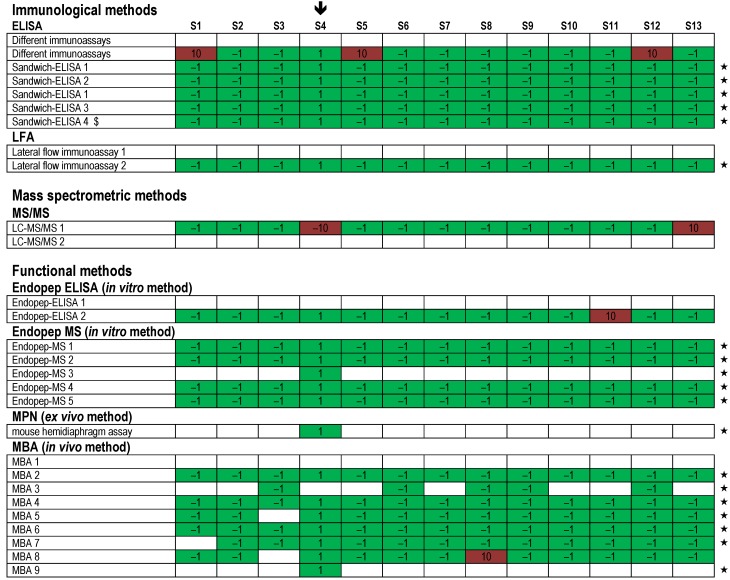
Qualitative results reported as “BoNT/E” for all 13 samples displayed by the different methods used. Samples containing BoNT/E are marked with a black arrow (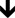). Each row indicates a qualitative result reported after using a particular method; several laboratories reported more than one method. Qualitative results reported by the participants were color-coded as described in Figure 5. Methods marked by an asterisk (★) delivered correct results for the samples analyzed. $, results have been taken from the laboratory’s quantitative reporting since they have, accidentally, not been reported qualitatively.

This analysis is displayed in Figure 5, Figure 6, Figure 7, Figure 8, Figure 9 and Figure 10, where each row indicates a qualitative result reported by a single method; several laboratories reported more than one method, therefore more than 23 rows are displayed in Figure 5, Figure 6, Figure 7, Figure 8, Figure 9 and Figure 10.

Figure 5 shows the comparison of qualitative results reported after using different methods for samples containing different concentrations of BoNT/A (from 1001 ng/mL to 0.5 ng/mL) in buffer. Based on the results reported for the very high concentration of BoNT/A in buffer (S12, 1001 ng/mL), 88.9% of results submitted were correct and 11.1% insufficient. For the sample with the high concentration of BoNT/A (S7, 100 ng/mL) all results were correct, for the intermediate concentration (S2, 9.9 ng/mL) 92.0% of results were correct and 8.0% insufficient, while for the moderate concentration of BoNT/A (0.5 ng/mL) 76.9% of results reported were correct and 23.1% insufficient. For the results submitted for the negative control sample (Figure 5b) containing buffer only, 96.0% of all results obtained by different methods were correct, 4.0% were partly correct and none was insufficient. So overall around 92%–100% of all results were correct when BoNT/A was present at high concentrations, while the percentage of correct results decreased to 76.9% when moderate concentrations were present. Considering that clinical or food samples often contain low ng/mL to pg/mL amounts of toxin, this basic result indicates that there is room for technical improvement to detect moderate and low concentrations of BoNT.

In Figure 6 the comparison is shown of qualitative results reported after using different methods for samples containing either about 100 ng/mL of BoNT/A (a) or 10 ng/mL of BoNT/A (b) in different matrices. Overall, the matrices offered posed no significant difficulties in analysis. It can be seen that 84.0 to 100% of results were correct for both concentrations, when toxin-spiked buffer, meat extract or milk were analyzed. Unexpectedly, the percentage of correct results dropped to 80.0% when toxin-spiked serum was analyzed. Taking into account that serum is the standard clinical matrix measured in suspicious botulism cases, the results offer potential for technical improvement.

Finally, Figure 7 displays qualitative results reported for different serotypes (BoNT/A, B and E) present at a concentration of around 10 ng/mL. In the direct comparison of samples containing one serotype in buffer, BoNT/A, B or E resulted in 92.0%, 88.0% or 95.5% correct results, respectively (Figure 7a). When sample S8 containing both BoNT/A and BoNT/B in equimolar concentrations (around 5 ng/mL) was analyzed, it turned out that 87.5% of results were correct for one serotype, BoNT/A, while only 75.0% of results were correct for the second serotype, BoNT/B (Figure 7b). The results suggest that a mixture of serotypes is more difficult to analyze than samples containing one serotype, indicating another starting point for method improvement.

Regarding the different technical approaches used by the 23 participating laboratories, a variety of methods were applied, combining different principles of detection and identification. An overview of qualitative results obtained by the different methods applied is given in Figure 8 for results reported as measurand “BoNT/A”, in Figure 9 for measurand “BoNT/B” and in Figure 10 for measurand “BoNT/E”, respectively.

Several laboratories used immunological detection methods: four different ELISA approaches, two different LFA approaches and several combinations of different immunoassays were reported. As shown in Figure 8 and Figure 10, all four ELISA approaches resulted in correct results for BoNT/A and BoNT/E on all 13 samples, while for BoNT/B flaws occurred using ELISA 1 and ELISA 4 on single samples (Figure 9). In contrast, ELISA 2 and ELISA 3 delivered correct results for all samples and all three analytes. Surprisingly, one of the LFAs (LFA 2) was able to identify correctly all samples for the measured serotypes BoNT/A, BoNT/B and BoNT/E, including the sample with the moderate concentration (S9, 0.5 ng/mL of BoNT/A), thus showing an exquisite detection limit of this on-site detection test compared to other LFAs with detection limits in the range of 5–50 ng/mL [64]. With respect to the immunological strategies applied in the PT, selected successful approaches are presented in more detail in a separate publication in this special issue of *Toxins* [65].

Two different LC-MS/MS approaches were used either on all samples or on selected, highly concentrated samples S5 (108 ng/mL of BoNT/A in meat extract), S7 (100 ng/mL in buffer) and S12 (1001 ng/mL of BoNT/A in buffer). Different sample preparation strategies were applied and combined with tryptic digest followed by LC-MS/MS analysis on different instruments. The approaches were successful in identifying several BoNT/A1-specific peptides from the LC and HC in samples S5, S7 and S12 that allow for unambiguous identification of the toxin including its subtype (Figure 8 and data not shown). Further technical adaptions, e.g., immunoaffinity enrichment of toxin, would be necessary to detect samples containing moderate to low concentrations of toxin.

In terms of functional testing, four different detection principles were applied by different laboratories: two Endopep-ELISA approaches, five Endopep-MS approaches, one MPN approach and nine classical MBA approaches (Figure 8, Figure 9 and Figure 10). Generally, the functional approaches resulted in good success rates for all samples and serotypes with only limited experimental flaws. Specifically, for each of the different functional *in vitro*, *ex vivo* or *in vivo* approaches, a number of protocols resulted in correct results (marked by an asterisk in Figure 8, Figure 9 and Figure 10). Endopep-ELISA 2 delivered correct results for all BoNT/A-, B- and E-containing samples except for sample S11 for which a false positive result was reported (Figure 10). Still, based on its overall performance, this approach was selected for a more detailed description [65]. Out of the five Endopep-MS approaches, four methods delivered correct results for all samples and all three analytes (Endopep-MS 1 to Endopep-MS 4). With respect to MS-based strategies including Endopep-MS protocols applied in the PT, successful approaches are presented in a separate manuscript in this special issue of *Toxins* [66]. Similarly good results were obtained by one laboratory applying an MPN assay, which again delivered qualitatively correct results for all samples and all three analytes measured. This assay is highlighted in [35].

The classical MBA was applied by nine laboratories, eight of which used the assay to perform serotyping using different mouse strains and different functionally blocking antibodies. One laboratory used MBA to determine BoNT-containing samples but did not differentiate serotypes (therefore the respective results given as MBA 1 appear white in Figure 8, Figure 9 and Figure 10; the reported data correspond to Figure 4, first row). Overall, results obtained by MBA were satisfying. Specifically, four protocols (MBA 4, MBA 6, MBA 7 and MBA 9) delivered correct results for all samples and analytes tested. In a previous inter-laboratory comparison of pharmaceutical BoNT products it has been shown that even for MBA as “gold standard method” and the analysis of highly pure BoNT preparations the results are variable depending on experimental parameters like sample preparation, the age and strain of mice or other factors [56,67]. After the PT, the feedback from laboratories performing MBA indicated that the laboratories experienced the restricted sample volume (1 mL) and the number of samples provided (13) as challenging experimental conditions, while these issues were generally not seen as problems by the laboratories applying *in vitro* or *ex vivo* methods.

Based on the qualitative PT results reported by the 23 participating laboratories, good analytical practices can now be derived: to this end, the different technical approaches applied were grouped together according to their detection principle and statistically analyzed, thus providing an overview of success rates obtained for the different detection principles on all 13 samples. This analysis is useful for drawing some general conclusions on the methods applied, while it is important to keep in mind that different analytical protocols and tools have been used and that for each detection principle successful individual strategies have been identified in the PT (Figure 8, Figure 9 and Figure 10). Additionally, one should keep in mind that the methods were applied by a variable number of laboratories, so that statistics should not be overestimated (e.g., the MPN assay was applied by one laboratory only, whereas the MBA was applied by nine participants, and ELISA and Endopep-MS were used by at least five laboratories). Table 3 provides an overview of success rates obtained for the different detection principles on all 13 samples. Generally, the success rate of the methods applied ranged from 80% to 100%. For the two immunological methods ELISA and LFA the success rate for correct results was 93.3% and 90.4%, respectively, which is in the range of results reported in a previous study: In a national ring trial five samples—four toxin-positive ones and a negative one—were distributed to 57 laboratories to evaluate a set of four BoNT/A-, B-, E-, and F-specific ELISA kits, resulting in 86% correct results for all five samples [68]. In the current BoNT PT the lowest percentage of correct results (80%) was reached when using LC-MS/MS methods, presumably because of the lower sensitivity of this method compared to others. The *in vitro* functional method Endopep-ELISA showed a success rate (93.6%) similar to that of the classical sandwich ELISA (93.3%). Interestingly, for qualitative analysis the other two functional approaches, the Endopep-MS assay (97.3%) and the MPN assay (100%), turned out to be as successful as the *in vivo* functional method, the classical MBA (97.6%). Therefore, it can be concluded that both methods represent an excellent alternative for BoNT detection and serotyping compared to the classical MBA. Against the background that the MPN assay was applied by one laboratory only, future exercises with more participants applying this method have to show if this good result can be reproduced. While the Endopep-MS assay as an *in vitro* method does not require animal tissue at all, the MPN assay still uses animal tissues, but fully replaces animal experiments and requires fewer animals than an MBA. Depending on the tools used for toxin enrichment, the Endopep-MS assay is able to represent the catalytic and the receptor-binding activity of BoNT only [38]. In contrast, the MPN assay has the advantage of detecting all steps of BoNT activity—surface receptor binding, internalization of toxin into the neuron, translocation of catalytically active LC into the cytoplasm, proteolytic cleavage of SNARE proteins plus blockade of neurotransmitter release; hence inactivated, non-dangerous BoNT is not measured, in contrast to some of the *in vitro* methods [35]. So depending on the application field—either diagnostics of suspected botulism samples or potency determination of BoNT pharmaceuticals—some method might be technically more appropriate than another one.

With respect to good analytical practices, the PT showed that laboratories’ applying either a combination of complementary *in vitro* approaches (immunological, MS-based plus functional methods) or an appropriate individual functional approach (either MBA or MPN or Endopep-MS assay) delivered superior results. This information will be crucial in the future to develop recommended operating procedures and optimized workflows for the analysis of toxin-containing samples that will be supported internationally.

**Table 3:** Qualitative results of methods used for BoNT serotyping: overview of success rates obtained after using different methods for all samples. *

Method	Total Number of Results	Number of Laboratories	Correct Results	%	Total %
**ELISA**	312	7	correct positive	24.4	93.3
			correct negative	68.9	
**LFA**	52	2	correct positive	38.5	90.4
			correct negative	51.9	
**LC-MS/MS**	55	2	correct positive	10.9	80.0
			correct negative	69.1	
**Endopep-ELISA**	63	2	correct positive	34.9	93.6
			correct negative	58.7	
**Endopep-MS**	222	5	correct positive	27.5	97.3
			correct negative	69.8	
**MPN**	13	1	correct positive	100.0	100.0
			correct negative	0.0	
**MBA**	330	9	correct positive	26.1	97.6
			correct negative	71.5	

* Detailed classification of success rates for the different methods applied for BoNT serotyping. For each method the total number of results reported per method is indicated, the number of laboratories applying an individual method and the percentage of correct positive or correct negative results.

#### 2.2.2. Quantitative Results of the BoNT Proficiency Test

Independent of the qualitative reporting, the participating laboratories were asked to perform quantification of BoNT/A, BoNT/B or BoNT/E in the 13 samples and to report the results of two independent measurements in a dedicated Excel reporting file. Again, for quantification any method established and validated in the laboratories was admitted; if the laboratories planned to use different methods for quantification, they were asked to submit results in separate quantitative reporting sheets. In this context, some basic questions were asked regarding the scope of assay validation performed prior to the PT (e.g., detection limit of the method, coefficients of variation and measurement uncertainty) and the reference material used. The quantitative measurements reported by the participants were—as far as possible—evaluated statistically according to the recommendations by Thompson *et al.* [60] and Algorithm A of the international standard ISO 13528:2005 “Statistical methods for use in proficiency testing by inter-laboratory comparisons” [69].

Quantification of BoNT/A, BoNT/B and/or BoNT/E in the 13 samples was performed by 11 of 23 participating laboratories (48%). In order to assess and visualize quantitative results, *z*-scores were calculated according to the equation

$$z=\frac{x-x_{a}}{\sigma_{p}}$$

with *x* denoting the results reported by the participants, *x*_a_ the assigned concentration value determined by the organizing laboratory and σ_p_ the standard deviation for proficiency assessment, respectively (Table 2). *z*-scores (in the context of PTs) quantify the difference between an individual single or mean result and the assigned value in units of the standard deviation for proficiency assessment. This transformation is known as standardization; the standardized data set has a mean of zero (0) and a standard deviation (and variance) of one (1) if *x*_a_ and σ_p_ are the respective statistics of the empirical distribution of the data considered. A *z*-score of zero indicates an unbiased result with respect to the assigned value, a *z*-score of 1 is one standard deviation for proficiency assessment above the assigned value, a *z*-score of −1 is one standard deviation for proficiency assessment below the assigned value and so on. Provided that the data points are realizations of normally distributed random variables with mean *x*_a_ and standard deviation σ_p_ (*i.e.*, *x* ~ *N*(*x*_a_, σ_p_^2^), this is the model to which the results reported are compared), the *z*-scores represent realizations of random variables of the standard normal distribution (*i.e.*, *z* ~ *N*(0, 1)), where about 95% of *z*-scores will fall between −2 and +2 (the sign “−” or “+” of the score indicates a negative or positive deviation, respectively). According to Thompson *et al.* [60], scores in this range are commonly designated “acceptable” or “satisfactory”. Scores in the ranges −2 to −3 and +2 to +3 would be expected about once in 20, and scores in this class are sometimes designated “questionable”. A score outside the range from −3 to +3 would be very unusual and is taken to indicate that the cause of the event should be investigated and remedied, and Thompson *et al.* [60] suggest the phrase “requiring action” for such results.

**Figure 11:**
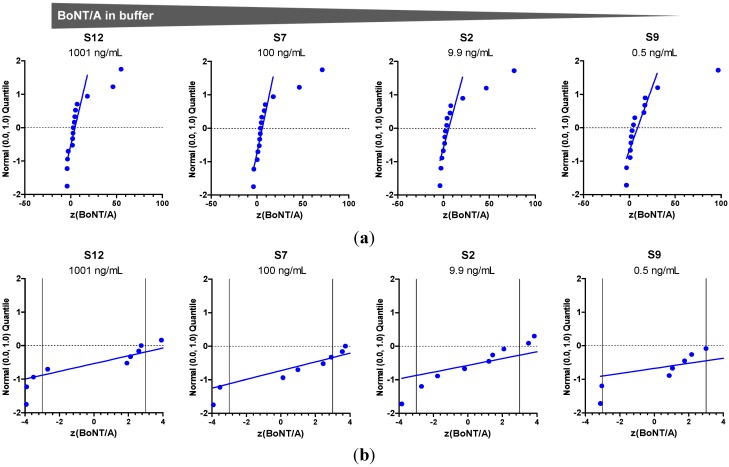
Normal probability plots of *z*-scores for quantification of BoNT/A. (**a**) Standard normal quantiles were plotted against the *z*-scores to visualize if scores (representing concentrations reported) were normally distributed. The analysis was done by considering all methods used to quantify the indicated samples. Each dot corresponds to one method used by one laboratory. The number of dots does not necessarily reflect the number of laboratories since several laboratories used more than one method to quantify the samples. (**b**) The magnified plot shows the data from (**a**) zoomed into *z*-scores from −4 to +4.

In the PT it turned out that precise quantification of BoNT/A, BoNT/B and/or BoNT/E was difficult, resulting in a substantial scatter of quantitative data. Exemplarily, the quantitative results provided by the participants for samples S12, S7, S2 and S9, containing different concentrations of BoNT/A in buffer starting from 1001 ng/mL to 0.5 ng/mL, are visualized in Figure 11. Normal probability plots of *z*-scores are displayed for all *z*-scores obtained (Figure 11a) and zoomed into the region between *z* = −4 and +4 (Figure 11b). *z*-scores of normally distributed concentration values with sample statistics *x*_a_ and σ_p_^2^ would lie along a straight line with slope 1 in these plots. Normal probability plots are used to display both the dispersion of a data set and the deviation of the empirical distribution from statistical normality. As can be seen in Figure 11, many of the quantitative data approximately follow the normal distribution of the model *x* ~ *N*(*x*_a_, σ_p_^2^), but some values are far off. Additionally, overall a positive bias of measured results from the assigned concentrations was observed, since the positive deviations of data reported were much wider than the negative deviations. This effect was seen independently of the methods used for quantification. The zoom-in in Figure 11b shows that four to six methods resulted in *z*-scores between −3 and +3, indicating that there is room for technical improvement. Still, taking into account that different reference materials were used in different laboratories, the results offer a sound basis for further steps in quality assurance.

In order to interpret the quantitative data obtained in the BoNT PT, the participants were asked to provide information on their BoNT standards used in the PT: standards from up to six different sources were used depending on the BoNT serotype (six different BoNT/A standards, four different BoNT/B standards and three BoNT/E standards, among them commercially available preparations and in-house materials of different quality). To understand basic factors influencing the quantitative data, the organizing laboratory compared two of the ELISAs applied by different participants and two different 150 kDa BoNT/A standards used in the PT. For the selected tools and assays, the results showed that the use of different ELISAs had no significant influence on the quantification of BoNT-containing samples, whereas the use of different reference standards clearly did: differences by a factor of two or more were obtained for the PT samples (data not shown). Based on this limited data that awaits further investigation, we speculate that the heterogeneity in quantification of BoNT/A, BoNT/B and BoNT/E in the PT samples is most likely due to the different reference materials used. Thus, quantification of BoNT can only be improved if a common certified reference standard becomes available—this is considered to be a priority aim for future research.

**Figure 12:**
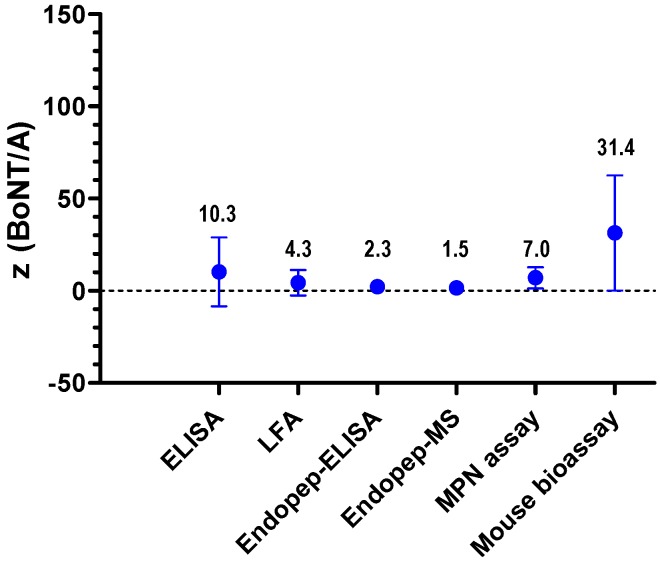
Accordance of methods used for quantification of BoNT/A in the BoNT PT. *z*-score means (points and figures) ± standard deviation as computed from the *z*-scores for methods specifically detecting BoNT/A. The analysis was done by considering all samples analyzed in the PT with the indicated methods. For the different methods applied in the PT, the following numbers of *z*-scores from single values reported were included: ELISA (140), LFA (38), Endopep-ELISA (20), Endopep-MS (20), MPN assay (20) and MBA (28).

In order to evaluate the different methods used in the PT for quantification of all samples, the accordance of methods was assessed for the different technical approaches. Figure 12 shows the *z*-score means (points and figures) and their standard deviations (error bars span mean ± SD) as computed from the *z*-scores. The *z*-score means offer a guide to assess the mean closeness of a method to the assigned concentration if applied by a number of laboratories to a number of samples, and the corresponding standard deviation measures the variation of the *z*-scores among the respective samples and laboratories. For *z*(BoNT/A) sufficient and comparable numbers of cases were obtained to draw some general conclusions (Figure 12): The *z*-score means vary from 1.5 to 31.4. Endopep-ELISA and Endopep-MS assay showed an acceptable mean closeness (absolute *z*-score < 2.5) with good accordance between the *z*-scores means among samples and laboratories (low standard deviation).

The *in vivo* method MBA showed the highest *z*-score mean (31.4) compared to the other methods, with less accordance between the *z*-scores among samples and laboratories (high standard deviation). The immunological methods, ELISA and LFA, as well as the MPN assay delivered intermediate results between the *in vitro* functional methods and the *in vivo* functional method.

The comparison of the different methods applied for quantification of BoNT/A showed the lowest accordance between the *z*-scores among samples and laboratories for the *in vivo* method MBA. This result might not be surprising, since previous work has already shown difficulties in quantification of BoNT activity using this *in vivo* assay [56]. Apart from the influence of different experimental factors on the actual performance of the MBA, the low accordance of data probably also reflects a more general difficulty in BoNT quantification which is related to the different units that can be used: quantification can either be based on (i) protein content, expressed e.g., in ng/mL, and/or based on (ii) biological activity, measured, e.g., in mouse LD_50_/mL. Laboratories using MBA or/and Endopep-MS assay for quantification measured the 13 PT samples in LD_50_/mL and calculated back into protein content based on literature data or own validation studies. However, in this calculation attention has to be paid on (i) the purity and specific activity of the reference material; (ii) the proportion of denatured, truncated, single chain or otherwise non-functional toxin in the reference material; (iii) the serotype and the subtype of toxin analyzed and, finally, (iv) the occurrence of toxin (pure di-chain toxin, M-PTC, L-PTC). Most of these factors are unknown for the in-house or commercially available reference materials used. Strictly speaking, the conversion of activity into protein content can only be applied for one defined preparation of BoNT where the above-mentioned factors are known. Also, the type of assay used to determine activity *in vivo*, *ex vivo* or *in vitro* plays an important role, as it is already known that different functional read-outs might result in different activities [6,68,70,71]. Also, species-specific effects on toxicity have been described and should be taken into consideration [72,73].

It cannot be ruled out that the low accordance between *z*-scores among samples and laboratories detected by MBA (Figure 12) could be due to a partial loss of functional activity of the toxins used to spike PT samples rather than true assay variation. In the stability study (Figure 2), quantitative ELISA were used to demonstrate sample stability based on (i) the high throughput of the method (130 samples had to be precisely quantitated on a single day); (ii) the high precision of the method; and finally (iii) the legal restraints in EU to sacrifice >13,000 mice for activity measurement to serve a diagnostic PT [74]. Against this background, we explicitly did not ask for determining functional activity, but to report on the samples’ protein content in ng/mL. In case of significant instabilities of the recombinant materials used to spike PT samples we would have expected that the 17 laboratories using different functional approaches (MBA, MPN assay, Endopep-MS, Endopep-ELISA) would have reported more diverse quantitative data. This was, however, not the case except for those laboratories using MBA. In the absence of a final proof, we think on the basis of available data that the stability of the recombinant material is satisfactory. This finding would be in line with results demonstrated for the pharmaceutical product incobotulinumtoxin A (Merz Pharma, Frankfurt am Main, Germany) comprising only the pure 150 kDa BoNT/A as active ingredient which displays a shelf-life of 3–4 years at room temperature [75]. In any case, on the way towards certified reference materials, future work has to address the formulation of toxins as well as more extensive stability studies.

When comparing different assay formats among each other, the molecular variability within the BoNT family with more than 40 known subtypes has to be considered [62,76]. The challenge of BoNT subtypes has not yet been addressed in this very first international BoNT PT, but surely will be an issue in future exercises. The large variability poses a challenge to genetic and immunological detection approaches. Basically, assays which rely on antibodies either for immunoenrichment or detection have to be validated on all known subtypes or sequence variants unless the exact antigenic epitope is known. However, such comprehensive analysis has been difficult to conduct due to the restricted access to all known subtypes. Also, the results obtained in this exercise are valid for the selected subtypes used to spike samples and have to be reviewed in future exercises on a broader panel of subtypes. On the other hand, for certain functional assays like MBA or MPN assay sequence variation does not impair detection of functional activity as long as the BoNT-subtype/variant is effective in the underlying species (e.g., human, mouse, rat). In an ideal situation, functional methods would deliver qualitatively identical results—quantitation of activity, however, can result in different units of activities which are method dependent. A more extensive discussion on this point can be found in Weisemann *et al.* [59] in this special issue of *Toxins*.

Generally, for the different application fields—from the health, food and security sectors to pharmaceutical industry—the specific biological activity of BoNT is the most informative measure on the quality of a given BoNT preparation; however, it is the most difficult one to standardize. In contrast, measurement of precise protein content as sum of active and inactive BoNT can be better standardized, e.g., by amino acid analysis [77]. With respect to future efforts to improve BoNT quantification in international laboratories, precise and accurate BoNT quantification requires appropriate certified reference materials with defined specific biological activity AND protein content that would be available to expert laboratories worldwide. These reference materials should be characterized with a broad range of different analytical methods, considering and extending the work that has been performed in the EQuATox project.

## 3. Experimental Section

### 3.1. Preparation of PT Samples

The BoNT preparations used in this PT to spike buffer and complex matrices are described in more detail in [59]. Briefly, as prototypic neurotoxins full-length BoNT/A1, BoNT/B1 and BoNT/E1 were produced by recombinant expression in K12 *E. coli* strains and isolated by fused affinity tags which were removed due to thrombin recognition site. To obtain maximum activity, posttranslational proteolytic activation of single chain BoNT was performed by hydrolyzing a thrombin recognition motif inserted into the sequence of the loop region connecting LC and HC. From the comprehensive characterization of the recombinant BoNTs produced it was concluded that BoNT/A1 was 99.9% pure and between 97% and 99% activated into LC and HC; BoNT/B1 was 96% pure and 100% activated; and finally BoNT/E1 was 93% pure and between 92 and 95% activated [59].

Protein concentration was determined by validated amino acid analysis [77]. The biological activity expressed as LD_50_ was determined by mouse bioassay (BoNT/A1, 1.43 × 10^8^ LD_50_/mg; BoNT/B1, 4.95 × 10^7^ LD_50_/mg; BoNT/E1, 4.08 × 10^7^ LD_50_/mg) [59]. Throughout this manuscript, the recombinant BoNT preparations are abbreviated BoNT/A, BoNT/B and BoNT/E, keeping in mind that subtype 1 has been used.

As complex food matrices ultra-high temperature (UHT) semi-skimmed milk and minced meat were purchased from a local retail store. Milk was opened under sterile conditions and spiked with a defined amount of BoNT as indicated in Table 1. A particle-free meat extract was prepared by extracting 30 g of minced meat from pork and beef (1:1) with 270 mL of 0.1% BSA/PBS buffer (pH 6). The meat was removed by centrifugation and the supernatant was autoclaved, filtrated and spiked with a defined amount of BoNT as indicated in Table 1. The spiked liquid food matrices were analyzed and quantitated for their BoNT concentration by sandwich ELISA without any further sample preparation. Human serum was selected as typical clinical sample in cases of suspected botulism and was collected from anonymous donors without demographic information, pooled prior to use and shown to be free of BoNT/A, B and E by sandwich ELISA. All three complex matrices as well as buffer (0.1% BSA/PBS) were spiked with defined amounts of BoNT/A, BoNT/B or BoNT/E as indicated in Table 1, and the amount of spiked toxin was quantified by sandwich ELISA specific for the different serotypes (Section 3.3).

### 3.2. Stability and Homogeneity Testing

In order to demonstrate sample stability during the PT test period (set to four weeks), 10 aliquots of each sample S1 to S13 were prepared by spiking of the matrices with BoNT/A, BoNT/B or BoNT/E as indicated in Table 1 and used prior to the actual PT for stability testing: five aliquots were stored for four weeks at −80 °C, and five aliquots were stored for four weeks at 4 °C for comparison (total number of aliquots: 130). After storage at the indicated condition the samples were frozen at −80 °C until analysis. All sample sets were analyzed simultaneously on a single day by different BoNT ELISAs corresponding to the serotype contained in a sample: an ELISA detecting BoNT/A for samples S1, S2, S4, S5, S7, S8, S9, S10, S11, S12 and S13; a BoNT/B-specific ELISA for samples S6 and S8 and an ELISA specific for BoNT/E (S4).

BoNT/A, BoNT/B and BoNT/E ELISAs were performed as described in Section 3.3. For analysis, all BoNT/A-containing samples were diluted to a concentration of 0.1 ng/mL which is in the linear range of the respective ELISA close to the EC_50_ value. Along the same line, for the BoNT/B-ELISA samples containing BoNT/B were diluted to a concentration of 0.3 ng/mL before analysis, and for analysis by BoNT/E-ELISA the sample containing BoNT/E was diluted to a concentration of 2 ng/mL.

For statistical analysis of ELISA results, outlying values were identified by Grubbs tests (R package “outliers [78,79]”) and excluded from the fitting of linear models with storage conditions as fixed effects and *post hoc* Dunnett tests with storage condition 4 weeks at −80 °C as control group, using SYSTAT 13 (SYSTAT Software Inc., Chicago, IL, USA).

For homogeneity testing, 40 aliquots of each sample S1 to S13 were prepared as before, and 10 randomly selected aliquots were used for homogeneity testing. Homogeneity of each test material was assessed according to Thompson *et al.* [60] and ISO/IEC 17043:2012 [61] on the basis of absorbance values at 450 nm obtained by sandwich ELISA and the deduced concentrations on the 10 test portions of each sample. The 10 randomly selected test aliquots of each sample were analyzed in duplicate in two independent experiments using BoNT/A-, BoNT/B- or BoNT/E-specific ELISA, respectively. The ELISAs were performed as described in Section 3.3.

The statistical analysis of ELISA data identified one obvious outlier which was excluded from the analysis according to Thompson *et al.* [60], consisting of Cochran tests to assess homogeneity of variances and the estimation of the variance components related to sampling and analytical variance, respectively. As the experiment was more sophisticated than that described in [60], factorial linear mixed models were set up to fit the data and to provide (robust) estimates of the sampling standard deviations and the analytical standard deviations, respectively, using the R package “robustlmm” [80]. Confidence intervals of the sampling variances were obtained by fitting the same models with SYSTAT 13 and assessed according to Recommendation 8. Furthermore, the analytical results of the homogeneity study were evaluated by the robust algorithm according to ISO 5725-5:1998 [63] to compute the nominal concentrations (using R package “metrology” [81]) which were finally used as *assigned values* in the calculation of *z*-scores.

Additionally, for the quantitative analysis of PT results reported by the participants, the standard deviation for proficiency assessment σ_p_ was calculated assuming a normal variate 0.95 confidence interval of [*x*_a_ − 0.5*x*_a_; *x*_a_ + 0.5*x*_a_] (corresponding to a reproducibility limit of 0.5*x*_a_), *i.e.*, σ_p_ = 0.5*x*_a_/1.96 = 0.255*x*_a_. As there are no “true” values or certified reference materials available, this was the choice made on the basis of the rule that inter-laboratory reproducibility limits are very often about twice the repeatability limits. The latter was assumed to be about 25% of the concentration, as was supported later by the experience in this PT (Table 2).

### 3.3. Amplified Sandwich ELISAs Specific for BoNT/A, B and E

Sandwich ELISAs specifically detecting BoNT/A, B or E were performed as described elsewhere [48,50,82] using mAb and pAb indicated in Table 4. BoNT/E-specific antibodies were generated similar to BoNT/A- and BoNT/B-specific reagents described in [48]. Briefly, Nunc MaxiSorp microtiter plates (Thermo Scientific, Brunswick, Germany) were coated with primary mAb (10 μg/mL) in 50 μL of PBS overnight at 4 °C and blocked with casein buffer (Senova, Jena, Germany) for 1 h at room temperature. After washing, 50 μL of toxin-containing solution was added in serial dilutions starting from 500 ng/mL to 6.4 pg/mL in assay buffer (PBS, 0.1% BSA; Sigma-Aldrich, Munich, Germany) as standard curve and incubated for 2 h at room temperature. The sandwich ELISA was developed by incubation with biotin-labelled secondary antibody diluted in casein buffer (1 h, room temperature), followed by a washing step and detection with Streptavidin-PolyHRP40 conjugate (0.5 ng/mL, Senova, Jena, Germany). After a washing step color reaction was developed by adding substrate 3,3′,5,5′-tetramethylbenzidine (TMB; SeramunBlau slow, Seramun Diagnostika, Heidesee, Germany). Reaction was stopped by 0.25 M sulfuric acid.

**Table 4:** Sandwich ELISAs specifically detecting BoNT/A, B or E. *

Antigen	Primary Ab, Name (Specificity)	Description [Ref.]	Secondary Ab, Name (Specificity)	Description [Ref.]
BoNT/A	A1688 (H_N_)	mAb [48,50]	HcA78 (H_C_)	mAb [82]
BoNT/B	B279 (LC)	mAb [48,50]	Botulism antitoxin Behring (LC, HC)	pAb: horse-anti BoNT/A, B, E, F(ab)_2_ fragments; Novartis, CH
BoNT/E	KE97 (LC, HC)	purified pAb from rabbit [65]	E136 (H_N_)	mAb [65]

* The domain recognized by the different antibodies is indicated with LC = light chain, H_N_ = *N*-terminal domain (H_N_) and *C*-terminal domain (H_C_) of the heavy chain (HC) of BoNT.

### 3.4. Statistical Analysis and Data Visualization

Qualitative responses (categories “0”, “1” and “n.a.”, indicating “absent”, “present” and “not analyzed/not available”, respectively) were compared to the correct answers by simple algorithms coding auxiliary variables representing the “success”: 1 for correct positive, −1 for correct negative, 10 for false positive, and −10 for false negative. The latter coding of single responses leads to mean values 4.5 and −4.5 if one of two reported results is false positive or false negative, respectively. For dichotomic grouping (correct/false) it was sufficient to use codes 1 and 0, respectively. On the basis of these categorical auxiliary variables, the success rates were obtained by frequency tabulation or by computing the means of the (0/1)-coded auxiliary variables grouped by the categories of interest (e.g., grouped by method). The resulting data tables were exported from SYSTAT to Excel^®^ and color-coded by conditional color formatting of each cell of the auxiliary variables.

Quantitative measurements reported by the participants were statistically evaluated according to the recommendations of Thompson *et al.* [60] and Algorithm A of the international standard ISO 13528:2005 [69] which is identical to the robust algorithm according to ISO 5725-5:1998 [45]. Robust algorithms of the R package “metrology” were used to compute the assigned concentrations. z-scores were obtained on the basis of these assigned values (Table 2) and the respective standard deviations for proficiency assessment (Section 2.1). Normal probability plots of the *z*-scores were produced by commercial software (SYSTAT 13) in order to visualize the empirical distributions of the results reported, as compared to the model implicitly set as normal distribution with mean x_a_ and variance σ_p_^2^, *i.e.*, *x* ~ *N*(*x*_a_, σ_p_^2^). Assessment of the accordance of methods was based on the arithmetic means of the individual *z*-scores as shown in Figure 12.

## 4. Conclusions

The aim of the BoNT PT conducted in the framework of the EQuATox project was to provide an overview and evaluation of existing methods for screening, detection and identification of BoNTs pathogenic to human among 23 participating laboratories from EU-28 and beyond. The exercise was the very first of its kind to explore qualitative and quantitative detection capabilities in international expert laboratories from the health, food and security sector. The 13 samples included BoNT/A, B and E in buffer, food and clinical matrices and were offered at restricted volume (1 mL), taking into account that in real botulism cases the available material is usually limited. The results highlight the *status quo* of detection capabilities which is—with 95% qualitatively correct results reported—generally good. In detail, potential for further technical improvement was identified with respect to: (i) sensitivity of methods; (ii) specificity of methods (serotyping) and (iii) matrix interference. Samples containing BoNT at low concentrations in pure solution or in matrix as well as serotype distinction were the most challenging tasks. A variety of methods were used by the participants, combining different detection principles. Generally, for all different technical approaches used by the participants, individual strategies were identified that delivered qualitatively correct results (as sum of good tools, good protocols, good experimental performance, so overall good proficiency). Two different sandwich-ELISAs and one LFA were identified that provided correct results for all samples and all serotypes down to the sub-ng/mL level, therefore these methods represent useful screening techniques and are presented in more detail together with a successful Endopep-ELISA approach in a separate manuscript in this issue of *Toxins* [65]. One LC-MS/MS method delivered correct results for toxin-containing samples in the ng/mL range and was suitable for the identification of the toxin subtype. As to MS-based strategies including functional approaches, four successful, highly sensitive Endopep-MS assay protocols were identified, and selected approaches are presented in a separate manuscript in this special issue of *Toxins* [66]. With respect to functional testing and in light of the new EU legislation on animal testing [74], there is an urgent need to replace the mouse bioassay as “gold standard” of BoNT detection. In this respect, the BoNT PT provided the valuable information that both the Endopep-MS assay [66] and the MPN assay [35] delivered results qualitatively similar to those of the MBA, but rendered superior quantitation of BoNT. Also, an Endopep-ELISA approach delivered convincing results, therefore this method should also be further explored as replacement method [65].

Generally, with respect to good analytical practices identified in the PT, it turned out that laboratories applying either a combination of complementary *in vitro* approaches (immunological, MS-based plus functional methods) or an appropriate individual functional approach (either MBA or MPN or Endopep-MS assay) delivered superior results. Based on this information, future efforts will address the development of recommended operating procedures and optimized workflows for the analysis of BoNT-containing samples—ideally this would be done in the context of a consolidated international EQuATox network. In technical terms, it has to be considered that the different methods vary with respect to trueness, precision, sensitivity, specificity and discriminative power, and that for all methods a dedicated validation is necessary to obtain reliable results. Still, the information generated in this PT will be very helpful for the decision of which methods can be used and combined in order to get preliminary, confirmed or unambiguous results for a BoNT-containing sample. Depending on the specific requirements of the application field (public health, forensic analysis or pharmaceutical industry), different methods or combinations of techniques might be selected as appropriate. To this end, the different method-papers in this special issue of *Toxins* provide contact points for laboratories seeking experimental advice.

In terms of quantification of BoNT, a substantial scatter of quantitative data was observed in the PT. Most methods provided results in the correct order of magnitude, but some *z*-scores were out of the range of −3 and +3, indicating that there is significant room for technical improvement. Comparative experiments in the organizing laboratory led to the conclusion that this scattering is very likely due to the different reference materials used in expert laboratories. Therefore, future technical improvement in quantification of BoNT will require appropriate certified reference materials with defined specific biological activity AND protein content that would be available to international expert laboratories. These reference materials should be useful for advances in quality assurance in all different sectors of BoNT research, the health, food and security sectors as well as pharmaceutical industries. In this context, the BoNT PT has provided a solid basis and a starting point for future advancement. Future challenges will address more difficult technical tasks such as detection and quantification of different/rare BoNT sero- and subtypes, pure di-chain toxin *versus* different complex forms and the analysis of more complex real sample matrices. To this end, the international discussions will have to focus on the general requirements regarding the level of harmonization or standardization that is useful and necessary in the different application fields to meet public health, clinical laboratory and forensic needs supporting efficient management decisions.
